# High-Risk Congenital Heart Disease in Pregnancy

**DOI:** 10.14797/mdcvj.1306

**Published:** 2024-03-14

**Authors:** Saurabh Rajpal, Carla P. Rodriguez

**Affiliations:** 1 The Ohio State University Wexner Medical Center, Columbus, Ohio, US; 2 Nationwide Children’s Hospital, Columbus, Ohio, US; 3 Boston Children’s Hospital and Brigham and Women’s Hospital, Harvard Medical School, Boston, Massachusetts, US

**Keywords:** complex congenital heart disease, cardiac risk stratification in pregnancy, systemic right ventricle, Fontan circulation, cyanotic congenital heart disease

## Abstract

High-risk congenital heart disease (CHD) in pregnancy presents a complex clinical challenge. With improved medical care and increased survival rates, a growing population of adults with complex CHD are surviving to adulthood, including women of reproductive age. This chapter focuses on risk stratification and management of pregnant women with high-risk CHD, emphasizing the importance of considering both anatomical and physiological complexity. Maternal physiological changes, such as blood volume increase, cardiac output changes, and alterations in vascular resistance, can significantly impact high-risk CHD patients. Management of high-risk CHD in pregnancy necessitates a multidisciplinary approach and individualized care.

## Introduction

Due to improved care and increased survival, there is a growing population of adults with complex congenital heart disease (CHD), including women of reproductive age desiring pregnancy. Peripartum intensive care unit admissions have increased, including admissions of women with CHD.^1^ Data from the European Society of Cardiology/European Observational Research Programme Registry Of Pregnancy And Cardiac disease (ROPAC), which is a prospective observational worldwide registry, shows that while women with complex CHD form about 15% of pregnant women with CHD, the risk of complications including mortality and heart failure is much higher in this cohort.^2^ Over the years, substantial efforts have been made to categorize maternal and fetal risks in individuals with CHD. In this review, we focus specifically on various high-risk CHDs, delving into their anatomical and physiological intricacies. It is important to note that the availability of primary source literature, especially randomized trials, is limited in this field, leading to recommendations primarily derived from registry data and expert consensus statements. Although we discuss a general framework for assessment and management, it is crucial to emphasize that the management of pregnancy in high-risk CHD is highly individualized and considers each patient’s unique anatomy, physiology, personal values, and beliefs.

## Risk Stratification

The modified World Health Organization (mWHO) risk classification, CARdiac disease in PREGnancy (CARPREG II), and the Zwangerschap bij Aangeboren HARtAfwijking (ZAHARA) risk scores address risk stratification in pregnant women with heart disease.^3,4,5^ When assessing pregnancy risk in women with complex CHD, one must consider both anatomical and physiological complexity, as highlighted in the 2018 American College of Cardiology/American Heart Association/Adult Congenital Heart Disease guidelines. A combination of these approaches may be used for individualized risk assessment in each patient.^6^

The mWHO classification system divides patients into four pregnancy risk classes (classes I–IV) based on their medical condition. For the purposes of this review, we will consider women in WHO Class III and IV as high-risk. Women in class III face a significant elevation in maternal mortality or severe morbidity, with a cardiac event rate ranging from 19% to 27%, while women in class IV carry an extremely high risk of maternal mortality or severe morbidity, with a cardiac event rate ranging from 40% to 100%, making pregnancy inadvisable. Tables 1 and 2 list congenital heart diseases with the highest pregnancy risk using the mWHO risk and CARPREG 2 risk assessment strategies. The CARPREG II risk score is based on 10 predictors (Table 3), each with an assigned weighted point score. The sum of points represents a risk score. The predicted risks for primary cardiac events stratified according to point score were 0 to 1 points (5%), 2 points (10%), 3 points (15%), 4 points (22%), and > 4 points (41%). It is noteworthy that while a majority (63.7%) of patients in the CARPREG II cohort had CHD, only a minority had complex CHD, and the authors indicate that this group may have been underrepresented. The ZAHARA risk score was developed particularly for women with CHD. This score included 8 predictors as shown in Table 4. The cardiac complications in percentage of total pregnancies based on ZAHARA risk score (0-13 points) were 0 to 0.5 (2.9%), 0.51 to 1.50 (7.5%), 1.51 to 2.50 (17.5%), 2.15 to 3.50 (43.1%) and > 3.51 (70%). Two predictors of this risk prediction rule are not mentioned in the CARPREG II study: the use of cardiac medication, and atrioventricular regurgitation. As mentioned above, a combination of these risk prediction models may need to be used in an individual patient to best characterize risk. For example, a patient who has mild systolic systemic right ventricular (RV) dysfunction and New York Heart Association (NYHA) class I would be classified as mWHO3. However, if the same patient also has a mechanical valve, she would still be characterized as mWHO3, but CARPREG II and ZAHARA would more accurately predict a higher risk. Similarly, the same patient with mild systolic systemic RV dysfunction and NYHA functional class I (mWHO 3) but with moderate to severe systemic atrioventricular valve regurgitation would indicate a patient at higher risk of cardiac events during pregnancy based on the ZAHARA score.

**Table 1:** Low- to intermediate-risk congenital heart disease in pregnancy. PDA: patent ductus arteriosus; ASD: atrial septal defect; VSD: ventricular septal defect


CONGENITAL HEART DISEASE	mWHO RISK (I, II AND II-III)

Uncomplicated small/mild pulmonary stenosis, PDA, mitral valve prolapse	I

Successfully repaired simple lesions (ASD, VSD, PDA, anomalous pulmonary venous drainage)	I

Unoperated ASD, VSD	II

Repaired tetralogy of Fallot	II

Repaired coarctation	II-III


**Table 2:** High-risk congenital heart disease in pregnancy. CHD: congenital heart disease; LV: left ventricle; mWHO: modified World Health Organization; NYHA: New York Heart Association; CARPREG: Cardiac Risk in Pregnancy Study; BAV: bicuspid aortic valve; HCTD: high risk connective tissue disease


CONGENITAL HEART DISEASE	PHYSIOLOGICAL VARIABLE	mWHO RISK (III vs IV)

Congenital severe aortic stenosis	Asymptomatic	III

	Symptomatic	IV

Unrepaired CHD	Hypoxemia (O_2_ < 94%)	III (degree of hypoxemia not addressed in mWHO or CARPREG 2)

	Severe hypoxemia	

Systemic right ventricle	Good or mildly reduced RV function	III

	Moderate to severely reduced RV function	IV

Fontan circulation	Well and uncomplicated	III

	Arrhythmias	IV

	Degree of hypoxemia	IV

	Moderate to severe systemic ventricular dysfunction	IV

	NYHA functional class	IV

Re-coarctation	Not severe	II-III

	Severe	IV

Any anatomically simple, moderate, or complex congenital heart disease	Pulmonary hypertension (including Eisenmenger syndrome)	IV

	Moderate LV dysfunction (EF 30-45%)	III (Unless classified as IV due to NYHA FC III-IV)

	Severe LV dysfunction (EF < 30%)	IV

	NYHA Functional Class II-IV	IV

	Arrhythmia	Not addressed in mWHO, use CARPREG for risk assessment

	Mechanical valve	III

Aortopathy	Moderate aortic dilatation (40-45 mm in Marfan syndrome or other HCTD; 45–50 mm in BAV, 20–25 mm/m^2^ in Turner syndrome)	III

	Severe aortic dilatation (> 45 mm in Marfan syndrome or other HCTD, > 50 mm in BAV, > 25 mm/m^2^ in Turner syndrome)	IV


**Table 3:** CARPREG II Risk Score. CARPREG: Cardiac Disease in Pregnancy Study; NYHA: New York Heart Association^5^


PREDICTOR	POINTS

Prior cardiac events or arrhythmias	3

Baseline NYHA III-IV or cyanosis*	3

Mechanical valve	3

Ventricular dysfunction	2

High-risk left-sided valve disease/left ventricular outflow tract obstruction	2

Pulmonary hypertension	2

Coronary artery disease	2

High-risk aortopathy	2

No prior cardiac intervention	1

Late pregnancy assessment	1


* NYHA risk groups include individuals with at least mild reduction in systemic ventricular systolic function (ejection fraction < 55%), high-risk valve lesions, or left ventricular outflow tract (LVOT) obstruction (such as aortic valve area < 1.5 cm^2^, subaortic gradient > 30 mm Hg, mitral valve area < 2 cm, or moderate to severe mitral regurgitation); those with mechanical valves; individuals with pulmonary hypertension (right ventricular systolic pressure ≥ 50 mm Hg in the absence of RVOT); those with high-risk aortopathy (including Marfan syndrome; bicuspid aortopathy with aortic dimension > 45 mm; Loeys-Dietz syndrome; vascular Ehlers-Danlos syndrome; or a history of aortic dissection or pseudoaneurysm); and individuals with coronary artery disease; defined as angiographically proven coronary obstruction or a history of myocardial infarction.

**Table 4:** ZAHARA Risk Score.^4^ ZAHARA: Zwangerschap bij Aangeboren HARtAfwijking; NYHA: New York Heart Association; AV: atrioventricular


PREDICTOR	POINTS

History of arrhythmias	1.5

Cardiac medications before pregnancy	1.5

NYHA class prior to pregnancy ≥ 2	0.75

Left heart obstruction (peak gradient > 50 mm hg or aortic valve area < 1 cm^2^)	2.5

Systemic AV valve regurgitation (moderate/severe)	0.75

Pulmonary AV valve regurgitation (moderate/severe)	0.75

Mechanical valve prosthesis	4.25

Cyanotic heart disease (corrected/uncorrected)	1.0


## Physiological Adaptations to Pregnancy with Special Considerations for Complex Congenital Heart Disease

For better understanding, the physiological adaptations to pregnancy can be divided into the antepartum, intrapartum, and postpartum periods. While details on the physiological adaptation to pregnancy are noted elsewhere in this compilation, we will discuss issues most relevant to high-risk CHD.

### Blood Volume and Anemia

Maternal blood volume increases to a maximum of roughly 40% by about 32 weeks aided by an increase in plasma volume and, to a lesser extent, an increase in red cell mass leading to what is called “physiologic anemia of pregnancy.”^7,8,9^ While this increase in blood volume is usually accompanied by a drop in systemic and pulmonary vascular resistance, which helps to accommodate the extra blood volume, this increase in blood volume in those with severe left ventricular dysfunction can lead to decompensated heart failure.^10,11^ This group of patients may have to be maintained on diuretics during pregnancy. Also, anemia and iron deficiency in pregnancy can be missed and remain untreated in those with cyanotic heart disease who tend to have higher hematocrit at baseline.^12^

### Cardiac Output

Cardiac output increases due to increases in stroke volume and heart rate in the early and later stages of pregnancy, respectively. It plateaus at about 24 weeks of pregnancy and is significantly affected by posture, with a significant drop in cardiac output and subsequently arterial pressure in the supine position.^13,14^ The increase in cardiac output in pregnancy can be blunted, especially in those with severe outflow tract obstruction (including severe coarctation), systemic ventricular dysfunction, pulmonary hypertension, and Fontan circulation.^15,16^ As a result, the ventricles may not be able to adequately increase cardiac output, leading to dyspnea, heart failure, or syncope. This group of patients may be advised to curtail physical activity but avoid immobility, which could put them at risk for thromboembolism. Avoiding over diuresis and maintaining adequate blood volume can be an important consideration in individual patients.

### Systemic Vascular Resistance

Systemic vascular resistance (SVR) drops to a nadir by 24 weeks and rises again during later pregnancy to reach preconception levels by term.^13,14^ This leads to about a 10 mm drop in diastolic blood pressure. This can be crucial in women with cyanotic heart disease where the decrease in systemic vascular resistance enhances the right-to-left shunt and hence hypoxemia.

### Pulmonary Vascular Resistance

Like SVR, pulmonary vascular resistance (PVR) decreases to a nadir by 24 weeks. This is important in accommodating the increase in cardiac output that occurs concomitantly and keeps the pulmonary arterial pressure within the normal range.^10,11^ This fall in PVR can be blunted or is absent in those with pulmonary arterial hypertension and Fontan circulation, which can lead to worsening pulmonary hypertension and/or the inability to augment cardiac output as usual in pregnancy.

### Hemostatic Considerations

Pregnancy is a hypercoagulable state due to multiple adaptations in the hemostatic pathway, including increased platelet aggregation and clotting factors and impaired venous return from the gravid uterus.^15^ This can be especially concerning in cyanotic heart disease as there may be an underlying right-to-left shunt that increases the risk of thromboembolism to the systemic circulation. Patients with cyanotic CHD are also at risk of increased bleeding with increased risk of maternal hemorrhage.^6,17^

### Intrapartum

Labor is associated with increases in cardiac output, systemic venous pressure, and blood pressure. These changes are attenuated if epidural anesthesia is used; however, these beneficial physiological changes may be limited in women with severe obstructive lesions and pulmonary hypertension, conditions that may affect blood pressure or lead to decompensated heart failure with pulmonary edema and low cardiac output.^6,15^ Also, the Valsalva maneuver, which is performed due to an urge to bear down when the fetus descends, can further reduce cardiac output and may be problematic in those with marginal cardiac output.^6,9^ For these patients, reducing the second stage of labor through assisted delivery methods such as forceps or vacuum extraction is advisable.

### Postpartum

After labor, cardiac output increases abruptly due to increased venous return resulting from uterine contractions and other factors. This is associated with increased systemic blood pressure after delivery and can lead to decompensated heart failure in those with marginal reserve. This effect is most pronounced within the first hour after delivery but normalizes to prepregnancy values by about 24 weeks.^9,18^ Blood loss is common after delivery and can lead to tachycardia and reduction in stroke volume.^19^

## Maternal and Neonatal Outcomes in Congenital Heart Disease

Pregnancy in CHD usually has a successful outcome in the modern era. However, maternal and fetal risk does increase with increases in CHD complexity.^6,9,15,16,20^ A meta-analysis including 27 studies with a total of 1,347 patients showed that while mortality was low overall, with only 9 reported deaths, most deaths occurred in those with complex CHD such as Eisenmenger’s syndrome and transposition of the great arteries. Arrhythmia, heart failure, and thromboembolic events were the other maternal outcomes more commonly seen in those with complex CHD. Hypertensive diseases of pregnancy and postpartum hemorrhage were seen across the spectrum of CHD.^20^

In this same metanalysis, 44 neonatal deaths were reported in 2,044 pregnancies. The highest rates of neonatal mortality were observed in women with Ebstein’s anomaly, Eisenmenger’s syndrome, Fontan, transposition of great arteries, and coarctation of aorta. Rates of Cesarean section, miscarriage, preterm delivery, and small for gestational age were also more frequent in those with severe lesions versus mild disease. Some other studies have reported higher perinatal events such as a higher preterm birth rate (10-12%), especially in those with complex CHD (22-65%) and a higher rate of small-for-gestational-age, respiratory distress syndrome, interventricular hemorrhage, and neonatal death (27.8%).^9,20,21^

## Management of High-risk Congenital Heart Disease in Pregnancy

Management of high-risk CHD is best done at a regional adult CHD center with intensive cardiac care units and a multidisciplinary team of obstetricians and anesthesiologists who are experienced in managing patients with CHD as well as a cardiothoracic or shock team if mechanical circulatory support is required.^6,15^ A care plan can be made based on the stage of pregnancy. While the first trimester is a time for baseline evaluation, the second trimester is when hemodynamic changes are at the maximum, therefore a careful physical exam should be done along with a detailed plan for delivery. Fetal echocardiogram is also performed in the second trimester. During the third trimester, patients with cyanosis or those with a history of heart failure could become symptomatic, and physical activity and employment may have to be curtailed. Table 5 is a step-by-step guide for general management of high-risk CHD in pregnancy. The sections below discuss outcomes and management in specific high-risk CHDs.

**Table 5:** Evaluation and management of women with high-risk congenial heart disease. mWHO: modified World Health Organization; NYHA: New York Heart: Association; CARPREG: Cardiac Risk in Pregnancy Study; ZAHARA: Zwangerschap bij Aangeboren HARtAfwijking; CHD: congenital heart disease


PRECONCEPTION COUNSELING		

	Estimate maternal risk	mWHO, CARPREG 2, ZAHARA

		Discuss environmental risk such as diabetes, smoking, teratogenic medications etc

	Discuss fetal risk	See section on outcomes

	Genetic counselling	Family history and prior pregnancy history

		Discuss risk of CHD in offspring of women with CHD (6% risk of CHD in offspring if mother has CHD, 3% if father has CHD; for autosomal dominant syndromes such as 22q11 deletion or Marfan syndrome, up to 50% risk)

		Offer genetic testing when index of suspicious is high either based on phenotype (syndromic) or otherwise (non-syndromic)

	Baseline testing	ECG, echocardiogram, cardiopulmonary exercise test, liver, kidney, and thyroid function tests. Consider cross-sectional imaging in vascular disease and when echocardiographic imaging is insufficient.

		Baseline O2 saturation, hemoglobin and coagulation studies, especially in cyanotic heart disease and those with thromboembolic risk

**DURING PREGNANCY**		

**First Trimester**	Establish care with multidisciplinary team at regional adult CHD center	Cardio-obstetrics, Adult congenital Heart Disease, Obstetrics, Maternal Fetal Medicine, Anesthesia

		Plan trimester-wise care and follow up

	Medication reconciliation	Ensure discontinuation of teratogenic medications

	Baseline testing	ECG, echocardiogram, baseline lab work as mentioned in preconception stage

	Discuss lifestyle issues	Physical activity, employment, mental health, thromboembolic risk

**Second Trimester**	Follow up visit	Consider repeat echocardiogram as hemodynamic changes are at maximum

		Fetal echocardiography

	Comprehensive plan for labor, delivery, and postpartum care	Service for Delivery: Labor and Delivery with or without Telemetry versus Cardiac Care Unit

		Sites for vascular access if hemodynamic monitoring in the peripartum period is planned.

		Anesthesia consults for those with possibly unstable hemodynamics, those with musculoskeletal deformities that may affect epidural placement and those with anticoagulation needs

		Cardiothoracic or Shock Team consult if mechanical circulatory support may be needed

		Social services consult if required for support

**Third Trimester**	Follow-up visit	Reassess physical activity, employment, mental health, thromboembolic risk

		Reassess and modify as needed plan for labor, delivery, and postpartum care

**INTRAPARTUM CARE**		

	Induction	Consider elective induction of labor ~39 weeks

	Position	Labor in right or left lateral tilt position

	Second stage	Avoid Valsalva or prolonged second stage of labor. Use vacuum or forceps delivery to shorten the second stage of labor

	Anesthesia	Cautious use of neuraxial anesthesia if cardiac output is preload dependent

		Preferred: epidural or combined spinal epidural analgesia using narcotic with a minimal dose of local anesthetic, least chance of reducing systemic vascular resistance and worsening right to left shunting in cyanotic patients

	Cesarean Section	Cesarean delivery is usually reserved for obstetric indications

		Filters in intravenous drips to avoid embolism in patients with right to left shunt

	Antibiotic prophylaxis	Reasonable to consider antibiotic prophylaxis in those with cyanotic heart disease

**POSTPARTUM**		

	Oxytocin	Not contraindicated as postpartum hemorrhage prevention is highly important but use cautiously as hypotension and tachycardia are possible side effects

	Close monitoring	Due to hemodynamic shifts first 24-48 hours are critical and close monitoring is warranted

	Thromboembolic risk	Early ambulation

	Postpartum visit	6-12 weeks to assess hemodynamic state

**ONGOING ADULT CONGENITAL HEART DISEASE FOLLOW-UP**		


### Left Ventricular Outflow Tract Obstruction

#### Congenital aortic stenosis

Left ventricular outflow tract obstruction (LVOT) can be valvular, supravalvular, or subvalvular. The most common is valvar aortic stenosis because of the bicuspid aortic valve.^22^ Although most patients with a bicuspid aortic valve would tolerate pregnancy well, some might present with significant aortic stenosis and dilation of the aorta. It is noteworthy that based on CARPREG II and ZAHARA scores, a peak gradient of over 50 mm Hg, a subaortic gradient > 30 mm Hg, or an aortic valve area < 1.5 cm^2^ is considered high risk in pregnancy as opposed to conventional valve guidelines.

Subvalvular and supravalvular aortic stenoses behave in similar fashion. Subvalvular aortic stenosis is the second most common type after valvar stenosis, and its presentation can range from a discrete fibrous membrane to a tunnel-like fibromuscular band. Between 14% and 27% of cases of repaired subvalvular aortic stenosis can present in adulthood with recurrence of LVOT or aortic valve regurgitation.^23^ According to CARPREG and WHO classification, patients with symptomatic severe LVOT should be counseled against pregnancy.^5^ Maternal cardiac morbidity during pregnancy is related to severity of obstruction and symptoms. The hemodynamic cardiovascular changes in pregnancy with increased cardiac output and drop in SVR could potentially put these patients at high risk for heart failure and arrhythmias. Patients with LVOT are at higher risk of small-for-gestational-age infants and premature births.^24^ As in severe valvar aortic stenosis, Cesarean section should be reserved for symptomatic LVOT, heart failure, or obstetric indications.

#### Coarctation of the Aorta

Most patients with repaired coarctation of the aorta (CoA) achieve adulthood without any significant complications from surgery. Depending on the type of repair, some patients can develop aortic aneurysm formation or re-coarctation. It has also been described that up to 30% of CoA cases can develop systemic hypertension, even without re-coarctation. Based on the mWHO stratification score for pregnancy in women with cardiovascular disease, pregnant women with repaired CoA are intermediate- to high-risk mWHO classes II and III. In contrast, women with unrepaired severe CoA are classified as mWHO class IV, which is severe risk. Preconceptionally, patients should have imaging studies such as an echocardiogram, computed tomography, or cardiac magnetic resonance imaging to assess aortic structure and dimensions.^22^ For women with coarctation of the aorta desiring pregnancy, the primary concern is the development of hypertension and preeclampsia.^25^ Women with unrepaired coarctation of the aorta are at risk of aortic dissection at the site of the narrowing and hypoperfusion of the placenta and limited fetal growth due to restricted blood supply to the lower part of the body. There are reports of a higher incidence of miscarriages in women with repaired coarctation versus controls.^26^ During pregnancy, close follow-up of blood pressure (careful 4 extremity blood pressure measurement) should be done at least every trimester. In cases of refractory blood pressure control or maternal or fetal hemodynamic compromise, invasive interventions such as re-coarctation stenting can be considered.^15^

### Systemic Right Ventricle with Biventricular Circulation

#### D-loop Transposition of Great Arteries

The surgical approach for patients with D-loop transposition of great arteries (D-TGA) has changed significantly over the past several decades—from the atrial switch (Mustard or Senning) procedure, where the right ventricle is the subaortic ventricle, to the arterial switch (Jatene) procedure, where the subaortic ventricle is the left ventricle. D-TGA patients in childbearing age can present with any of these two surgical procedures. The most recent data have shown that patients with atrial switch can tolerate pregnancy, with a low mortality but high morbidity. The most common cardiac complications are arrhythmias, heart failure, worsening of systemic right ventricular function, and tricuspid regurgitation. In addition, there is a high incidence of premature births and fetal loss.^27^ As in other cases, Cesarean section is reserved for patients with acute heart failure at the time of delivery.

**Figure 1:**
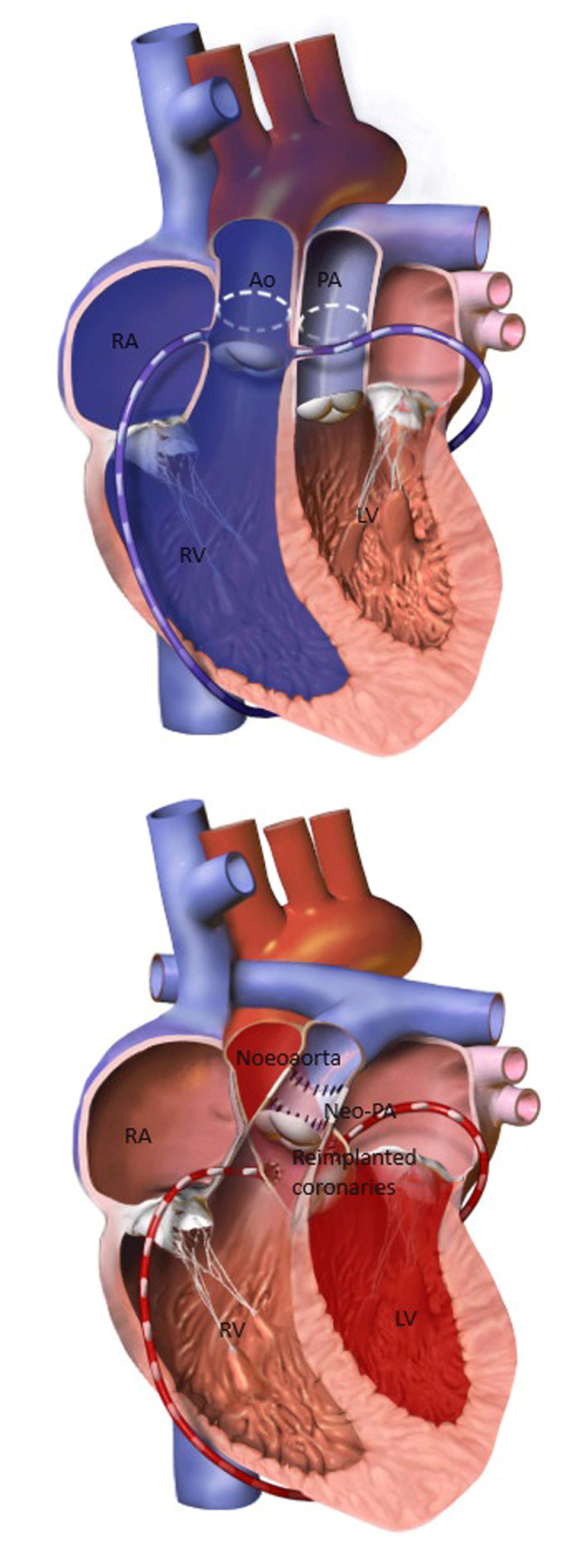
(Top) D-Loop transposition of great arteries; (Bottom) D-Loop transposition of great arteries, post arterial switch operation. Courtesy of Bruce Blausen (2014); *WikiJournal of Medicine*. RA: right atrium; RV: right ventricle; PA: pulmonary artery; Ao: aorta; LA: left atrium; LV: left ventricle

For patients with D-TGA who undergo the arterial switch procedure (Figure 1), the outcomes have been more favorable, with reports of a lower incidence of arrhythmias and heart failure events. Obstetric and neonatal complications are also less common than in cases of atrial switch.^2^ In some reported cases, D-TGA arterial switch patients have required C-section due to aortic dilation. It is important to rule out coronary disease using anatomical or physiological imaging in adulthood (preconception) as recommended in guidelines.^6^

#### L-loop Transposition of Great Arteries

**Figure 2:**
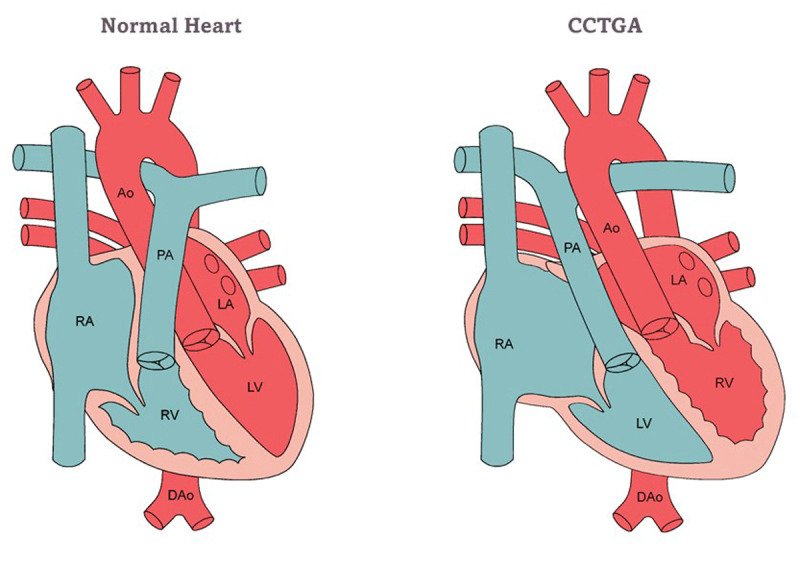
Congenitally corrected transposition of great arteries. Image Courtesy of the Adult Congenital Heart Association. RA: right atrium; RV: right ventricle; PA: pulmonary artery; Ao: aorta; LA: left atrium; LV: left ventricle; DAo: descending aorta

In a small case series of patients with congenitally corrected transposition of the great arteries (Figure 2), pregnancy is well tolerated. Even so, it comes with a higher risk of cardiovascular complications and fetal loss than the general population.^28^ Signs of heart failure before pregnancy and systemic right ventricular ejection fraction of < 40% have been identified as predictors of major adverse cardiac events during pregnancy.^2^

### Fontan Procedure and Single Ventricle Physiology

**Figure 3:**
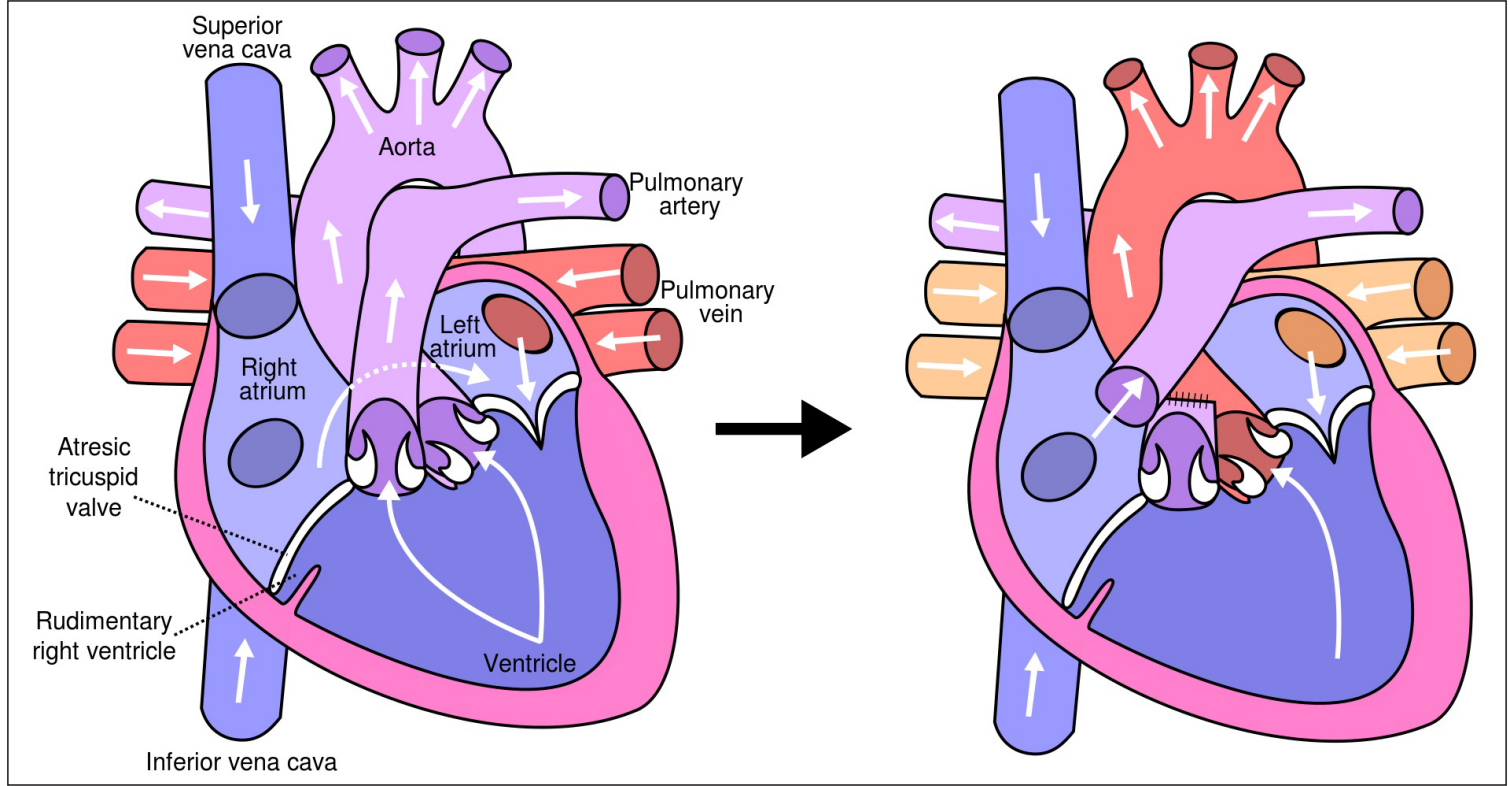
(Left) Diagram of the human heart with tricuspid atresia; (Right) Diagram of the human heart after Fontan procedure, via Wikimedia Commons.

Many individuals with single ventricle physiology who have undergone the Fontan procedure (Figure 3) can successfully reach adulthood and attain childbearing age.^6^ This patient cohort exhibits significant heterogeneity, primarily attributable to the nature of the underlying cardiac condition (single left ventricle versus single right ventricle) and the diverse iterations of the Fontan operation performed across different historical periods and geographical regions. Despite the availability of risk assessment scores that generally classify Fontan patients as high risk, practical risk evaluation remains highly personalized. A significant number of women with Fontan procedure may tolerate pregnancy well but with a higher risk of cardiac complications. Some research suggests that more recent iterations of the Fontan procedure may entail fewer pregnancy-related complications, emphasizing the potential need for a dedicated risk stratification score tailored to this specific patient population.^29^ It is imperative to underscore that the care of these patients should be exclusively entrusted to regional adult congenital heart disease centers.^6,15^

Arrhythmias and heart failure are the most common cardiovascular complications in women who have undergone the Fontan procedure. Supraventricular arrhythmias are the most common type of arrhythmia and tend to occur most commonly in the third trimester, while heart failure usually occurs in the postpartum period.^30^ There is also a high incidence of premature birth and miscarriages. Patients with Fontan procedure are also at risk of postpartum hemorrhage, which stems from several factors, including lower use of uterotonic agents, use of anticoagulation or antiplatelet agents, and underlying liver and thrombotic disorders.

### Cyanotic Congenital Heart Disease

**Figure 4:**
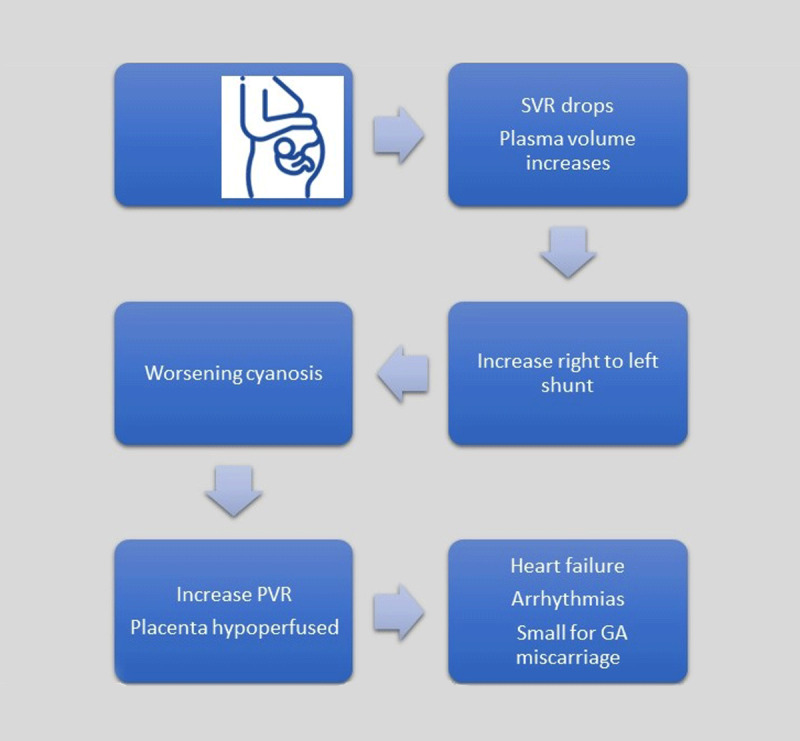
Hemodynamic changes in cyanotic patients during pregnancy. SVR: systemic vascular resistance; PVR: pulmonary vascular resistance; GA: gestational age

Despite anatomic complexity and high morbidity, patients with cyanotic CHD can reach adulthood and childbearing age. Cyanotic congenital heart disease constitutes a heterogeneous group of unrepaired shunt lesions, palliated surgeries, or single ventricle physiology. Patients with cyanotic congenital heart disease and normal pulmonary vascular resistance have restricted pulmonary blood flow. Some examples are unrepaired tetralogy of Fallot, Fontan procedure with venous-venous collaterals, and unrepaired Ebstein anomaly with atrial septal defect. Those with unrestricted pulmonary blood flow usually develop severe pulmonary hypertension and eventually Eisenmenger physiology. Some examples are atrioventricular septal defect and unrepaired patent ductus arteriosus.^31^ Secondary erythrocytosis and hyperviscosity are commonly observed in chronic cyanosis. During pregnancy, hemodilution improves hyperviscosity. Studies in cyanotic CHD through pregnancy have shown that cardiovascular complications depend on the type of cardiac defect and physiology of the patient (Figure 4).

#### Cyanotic Congenital Heart Disease Without Pulmonary Hypertension

Cyanotic patients with oxygen saturations < 90% at rest should be encouraged to use supplemental oxygen during pregnancy. The frequency of follow-up during pregnancy should be individualized, but close follow-up, serial N-terminal pro–B-type natriuretic peptide, and echocardiography are recommended. Heart failure is the most common severe complication during pregnancy. If a patient develops heart failure, strict bedrest, oxygen supplementation, and fluid management are usually advised.^32^ Bleeding and thromboembolism are the most common complications in the peripartum and postpartum period. There is also a high risk for fetal complications, including miscarriages, premature births, and low birth weight.^32^ Fetuses from cyanotic mothers also have a higher risk of CHD.^33^

#### Cyanotic Congenital Heart Disease with Pulmonary Hypertension

In this subgroup, the presence and severity of pulmonary hypertension determines maternal morbidity and mortality. Maternal mortality in patients with pulmonary hypertension has lowered in the past decades but is still as high as 30%.^31^ Most patients with pulmonary hypertension are advised against pregnancy, but if pregnancy occurs, they should be followed by a multidisciplinary team. As pregnancy evolves, cardiac output and pulmonary artery pressure both increase, but eventually the ventricle cannot keep up with the demands, and heart failure develops. In addition, the drop in SVR causes worsening hypoxemia due to increased right-to-left shunt, which also causes an increase in PVR. The most common complications found in cyanotic patients with pulmonary hypertension are right-side heart failure, pulmonary artery thrombosis, and pulmonary hypertensive crisis. These events mainly occur in the puerperium or the postpartum period.^15^ Ideally, in the preconception period, pulmonary hypertension medications should be revised and adjusted. The endothelin receptor antagonists, such as bosentan, are associated with embryopathy and should be discontinued before pregnancy.^34^

## Conclusion

High-risk CHD in pregnancy requires careful risk stratification, management, and monitoring. The physiological changes during pregnancy can have variable effects on patients with complex CHD, and a personalized approach is essential to optimize outcomes for both mother and baby. It is critical to recognize the challenges and complexities of managing pregnancy in these patients and provide appropriate care in specialized centers with expertise in CHD and obstetric care.

## Key Points

- The modified World Health Organization (mWHO) risk classification, CARdiac disease in PREGnancy (CARPREG II), and the Zwangerschap bij Aangeboren HARtAfwijking (ZAHARA) risk scores help stratify risk in pregnant women with heart disease.
- Maternal physiological changes, such as blood volume increase, cardiac output changes, and alterations in vascular resistance, can have varying effects on patients with high-risk CHD.
- Hemostatic considerations, including hypercoagulability, are crucial in women with cyanotic heart disease.
- Intrapartum challenges, including the Valsalva maneuver during the second stage of labor, require consideration and may necessitate assisted delivery methods.
- Abrupt changes in cardiac output can affect high-risk CHD patients after delivery, especially those with marginal reserves.
- Maternal and neonatal outcomes vary, with complex CHD associated with higher risk.
- Management of high-risk CHD in pregnancy necessitates a multidisciplinary approach, individualized care, and consideration of the stage of pregnancy.
